# Higher homocysteine and fibrinogen are associated with early-onset post-stroke depression in patients with acute ischemic stroke

**DOI:** 10.3389/fpsyt.2024.1371578

**Published:** 2024-06-28

**Authors:** Mingzhu Deng, Nina Zhou, Kangping Song, Zhen Wang, Wei Zhao, Jiayu Guo, Sufen Chen, Yangping Tong, Wei Xu, Fangyi Li

**Affiliations:** ^1^ Department of Neurology, Brain Hospital of Hunan Province, The Second People’s Hospital of Hunan Province, Changsha, Hunan, China; ^2^ Department of Neurology, The Affiliated Changsha Central Hospital, Hengyang Medical School, University of South China, Changsha, Hunan, China

**Keywords:** post-stroke depression, acute ischemic stroke, homocysteine, fibrinogen, inflammation

## Abstract

**Background:**

Post-stroke depression (PSD) is a well-established psychiatric complication following stroke. Nevertheless, the relationship between early-onset PSD and homocysteine (Hcy) or fibrinogen remains uncertain.

**Methods:**

Acute ischemic stroke (AIS) patients who met the established criteria were enrolled in this study. Early-onset PSD was diagnosed two weeks after the stroke. The severity of depressive symptoms was assessed by the Hamilton Depression Scale-17 items (HAMD-17), with patients scored ≥7 assigned to the early-onset PSD group. Spearman rank correlation analysis was employed to evaluate the associations between Hcy, fibrinogen, and HAMD scores across all patients. Logistic regression analysis was conducted to investigate the relationship between Hcy, fibrinogen, and early-onset PSD. Receiver operating characteristic curve (ROC) analysis was ASSDalso performed to detect the predictive ability of Hcy and fibrinogen for early-onset PSD.

**Results:**

Among the 380 recruited patients, a total of 106 (27.89%) patients were diagnosed with early-onset PSD. The univariate analysis suggested that patients in the PSD group had a higher admission National Institutes of Health Stroke Scale (NIHSS) score, modified Rankin Scale score (mRS), Hcy, and fibrinogen levels than patients in the non-PSD group (P<0.05). The logistic regression model indicated that Hcy (odds ratio [OR], 1.344; 95% confidence interval [CI] 1.209–1.494, P<0.001) and fibrinogen (OR, 1.57 6; 95% CI 1.302–1.985, P<0.001) were independently related to early-onset PSD. Area under curve (AUC) of Hcy, fibrinogen, and Hcy combined fibrinogen to predict early-onset PSD was 0.754, 0.698, and 0.803, respectively.

**Conclusion:**

This study indicates that Hcy and fibrinogen may be independent risk factors for early-onset PSD and can be used as predictive indicators for early-onset PSD.

## Introduction

Post-stroke depression (PSD) is a severe and frequent complication of mental disorders followed by stroke, with an estimated prevalence range from 18% to 33% (1, 2). The clinical manifestations of PSD are characterized by low mood, loss of interest, and even suicidal tendencies (3). PSD is associated with increased death rates and places additional burdens on both the affected individuals’ families and society as a whole (4). PSD can occur in different stages after stroke, early-onset PSD refers to patients exhibiting depression within two weeks after acute stroke onset (5, 6). Compared to late-onset PSD, early-onset PSD is characterized by a greater prevalence of depressive symptoms and is substantially associated with a greater chance of poor outcomes (7). Thus, it is of great value to detect predictive factor for early-onset PSD.

Inflammation plays a crucial role in the pathophysiology of PSD (8). Several studies have reported a strong correlation between Hcy levels and inflammation (9, 10). Fibrinogen is a common biomarker of inflammation (11, 12). Studies have shown that serum Hcy levels are elevated in patients with depression and correlate with the severity of depressive symptoms (13). Reducing Hcy might help alleviate anxiety and depression (14). In addition, previous studies have shown that elevated Hcy at admission is associated with PSD at 3 months after stroke (15). High-sensitivity C-reactive protein combined with Hcy can more accurately predict PSD at three months and one year after stroke (16, 17). Genetic factors may affect the serum levels of Hcy (18). The C677T is a non-synonymous variant, leading to a reduction in the activity of methylenetetrahydrofolate reductase (MTHFR) and folate levels and elevating serum Hcy levels (19, 20). MTHFR C677T mutations and folate deficiency could increase the risk of coronary heart disease and ischemic stroke in later life (21). A previous study found that the MTHFR C677T AG genotype and A allele increased the risk of PSD (22). Furthermore, a recent study showed that MTHFR C677T may exert an effect on PSD via mediating Hcy level (18). So far, the relationship between Hcy and early-onset PSD is still unclear. Based on the above research, we speculate that Hcy may be closely related to the occurrence and development of early-onset PSD.

Previous studies have found that patients with acute coronary syndrome revealed high levels of fibrinogen, and they were significantly higher among patients with anxiety and depressive disorders (23). In healthy individuals, psychological distress is also correlated with hypercoagulability (24). Both coagulation and fibrinolysis increase from baseline in response to acute stress, but coagulation increases more than fibrinolysis (25). Meanwhile, a high level of fibrinogen has been associated with depression 1- or 3-months post-stroke onset (26, 27). The underlying mechanism of how fibrinogen causes PSD remains unclear. Some researchers have proposed that fibrinogen might be a mediator between stroke severity and inflammatory response, the latter leading to depressive symptoms (26, 28). To date, there have been few studies concerning the relationship between fibrinogen and early-onset PSD.

At present, numerous risk factors, including gender, age, inflammatory cytokines, lesion localization, stroke severity, a history of depression, and symptomatic plaque enhancement, have been identified by researchers as being associated with PSD (2, 29). However, the relationship between Hcy and early-onset PSD remains unknown, and there have been few studies concerning the relationship between fibrinogen and early-onset PSD. In this study, we aimed to investigate the association of Hcy and fibrinogen with early-onset PSD and to explore whether Hcy and fibrinogen can serve as predictive indicators for early-onset PSD.

## Materials and methods

### Study design and participants

**Figure 1:**
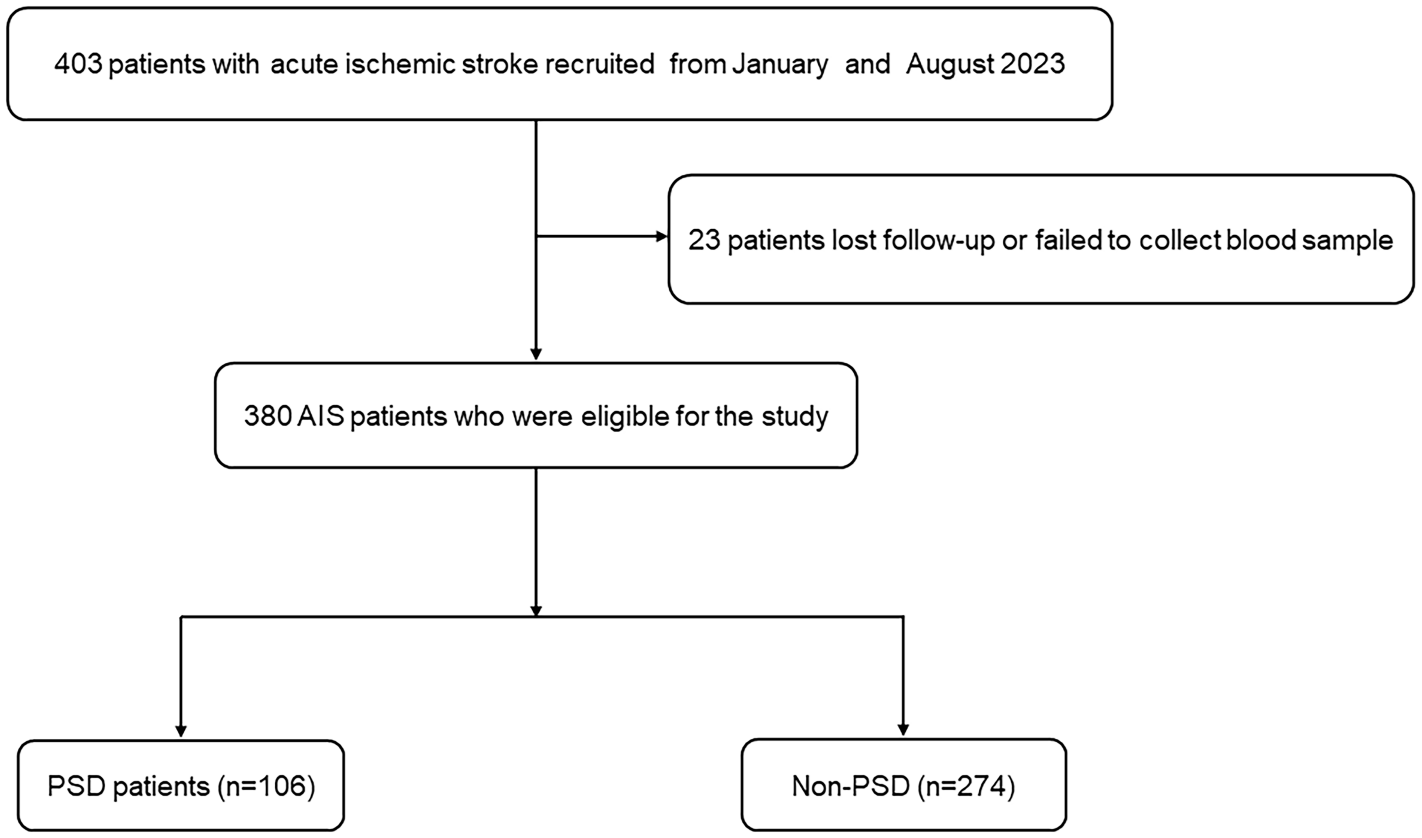
The study flow diagram. AIS, acute ischemic stroke; PSD, post-stroke depression.

Acute ischemic stroke (AIS) patients were prospectively recruited from the Affiliated Changsha Central Hospital, Hengyang Medical School, University of South China, between January and August 2023. This study was approved by the Ethics Committee of the Affiliated Changsha Central Hospital, Hengyang Medical School, University of South China. Eligible participants were enrolled in the final analysis if they met the following criteria. Inclusion criteria: (1) patients who fulfilled the diagnostic criteria for ischemic stroke as outlined in the Chinese guidelines for diagnosis and treatment of acute ischemic stroke 2018 (30), with AIS confirmed by computed tomography (CT) or magnetic resonance imaging (MRI) within 24 hours after admission; and (2) patients aged between 18 and 85 years; and (3) patients who were admitted to the hospital within 72 hours after the onset of stroke. Exclusion criteria: (1) patients with severe aphasia or dysarthria and a consciousness disorder that prevents them from completing evaluations and questionnaires; (2) patients with a pre-existing diagnosis of dementia or significant cognitive impairment prior to the stroke; (3) patients with severe heart, liver, or renal insufficiency; (4) patients who self-report having any psychiatric illness, including depression, or who were using psychotropic drugs prior to the stroke onset; (5) patients with medical histories of other central nervous system diseases such as Parkinson’s disease and epilepsy;(6) patients with malignant tumors that may potentially cause metabolic abnormalities; and (7) patients with nutritional disorders. There were 380 AIS patients recruited between January and August 2023 (**Figure 1**).

### Clinical characterization

All participants underwent standard evaluations of their age, sex, body mass index, vascular risk factors (hypertension, diabetes mellitus, atrial fibrillation, coronary artery disease, current drinking and smoking, and laboratory data) on the day of admission. Smoking was defined as a patient who had smoked continuously for 5 years with at least 10 cigarettes per day. Alcohol consumption was defined as a patient who had drunk continuously for 5 years with at least 20g ethanol per day. The National Institutes of Health Stroke Scale (NIHSS) was used to evaluate stroke severity at admission by trained neurologists. NIHSS scores were assessed within 24 h after admission. The Barthel Index (BI) scores were assessed at discharge. In addition, the functional outcome was also assessed according to the modified Rankin Scale (mRS) score at follow-up after 1 month. The lesion site and stroke subtype were determined by computed tomography, magnetic resonance, electrocardiogram, echocardiography, carotid ultrasonography, and transcranial doppler.

### Clinical assessment and subject grouping

According to the Diagnostic and Statistical Manual of Mental Disorders, 5th edition (DSM-V) (31), trained neurologists and psychiatrists diagnosed patients with PSD 2 weeks after stroke onset. The severity of depressive symptoms was evaluated with the Hamilton Depression Scale 17 items (HAMD-17) (32). According to the recommendation, HAMD-17 scores <7 mean normal condition (33), and patients with these scores were enrolled in the non-PSD group. Patients with HAMD-17 scores greater than or equal to 7 were included in the PSD group. A score of 7−17, 18−23, and more than 24 indicates mild depression, moderate depression, and severe depression, respectively (33–35). According to the scores obtained, patients were classified into the mild, moderate, or severe PSD groups.

### Blood Biomarker Examination.

Blood samples of all patients who within 72 hours after the onset of stroke were collected at 6–7 a.m. the day after fasting for at least 8 h. Two milliliters of EDTA-anticoagulated whole blood were used for routine blood tests (automated hematology analyzer, BZ6800, CHINA) that included white blood cell (WBC), neutrophils, and lymphocyte counts. Five milliliters of blood containing coagulant were used for a common biochemical examination (automatic analyzer, HITACHI 7600, JAPAN) that included creatinine (Cr), uric acid (UA), triglycerides (TG), total cholesterol (TC), high-density lipoprotein cholesterol (HDL-C), low-density lipoprotein cholesterol (LDL-C), homocysteine (Hcy), and fibrinogen. All the indicators were tested using commercial kits, which were operated by qualified professionals in accordance with the specifications.

### Statistical analysis

Data analysis was performed using SPSS 25.0 (IBM SPSS Statistics software, Version 25.0). Categorical variables were expressed as n (%), and normally distributed continuous variables were expressed as means (standard deviation, SD), otherwise, the data are presented as the median (interquartile range). Differences in baseline characteristics between groups were analyzed using the one-way ANOVA test or Mann-Whitney U test for continuous variables as well as the chi-squared test or Fisher’s exact test for categorical variables, as appropriate. We used the scatter plot to show the distribution of serum Hcy and fibrinogen levels among PSD of different severity. Analyses using the Spearman rank correlation were carried out to investigate the correlation that exists between the Hcy, fibrinogen, and HAMD scores in all patients. Binary logistic regression analysis for risk factors with PSD. A MedCalc 15.6.0 (MedCalc Software Acacialaan 22, B-8400 Ostend, Belgium) packet program was used to obtain a receiver operating characteristic (ROC) curve to test the overall discriminative ability of Hcy and fibrinogen for PSD. A two-tailed value of P<0.05 was considered significant.

## Results

### Clinical and demographic characteristics of non-PSD and early-onset PSD

**Figure 2:**
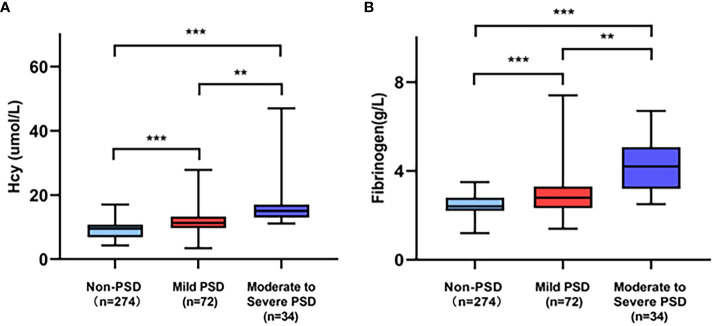
Comparison of serum Hcy **(A)** and fibrinogen **(B)** level among PSD of different severity. ***p<0.001, **p<0.01.

**Figure 3:**
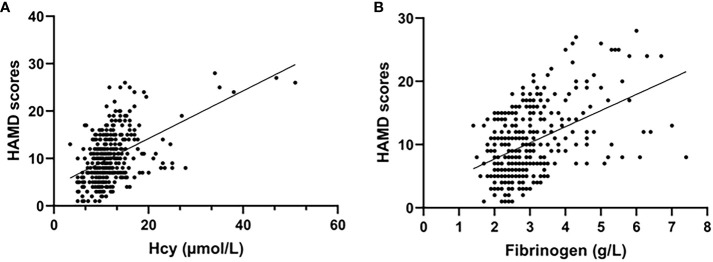
Correlation analysis between the plasma Hcy, fibrinogen and the HAMD scores in all patients. Scatter plots showing the plasma Hcy [r=0.441, p<0.001; **(A)**] and fibrinogen [r=0.407, P<0.001; **(B)**] were positively correlated with HAMD scores.

The demographic and clinical characteristics are comprehensively elaborated in **Table 1**. Early-onset PSD was observed in 106 patients (27.89%), and non-PSD was observed in 274 patients (72.11%), respectively. In the early-onset PSD group, current smoking (P=0.022), NIHSS score (P=0.004), mRS (P<0.001), HAMD-17score (P<0.001), Hcy (P<0.001) and fibrinogen (P<0.001) were significantly higher than those in the non-PSD group, while the BI score (P<0.001) was significantly lower than those in the non-PSD group. In addition, serum Hcy and fibrinogen levels were compared among early-onset PSD of different severity (**Figure 2**). Plasma Hcy (r=0.441, p<0.001) and fibrinogen (r=0.407, P<0.001) were positively correlated with HAMD scores (**Figure 3**).

**Table 1:** Characteristics of patients in the non-PSD and PSD groups.

Variable	Non-PSD (n=274)	PSD (n=106)	T/Z	*P*
Demographic characteristics				
Age, years	64.42 ± 11.22	66.15 ± 12.04	-1.249	0.212
Male, n (%)	211(77.01)	70(66.04)	4.774	0.029
BMI, kg/m^2^	23.69 ± 3.32	23.29 ± 3.56	1.283	0.202
Vascular risk factors, n (%)				
Hypertension	230(83.94)	82(77.36)	2.254	0.133
Diabetes mellitus	101(36.86)	35(33.02)	0.491	0.483
Coronary artery disease	49(17.88)	15(14.15)	0.760	0.383
Current smoking	148(54.01)	43(40.57)	5.530	0.019
Current drinking	66(24.09)	28(26.42)	0.222	0.637
Lesion location, n (%)			4.667	0.097
Anterior circulation	164(59.85)	74(69.81)		
Posterior circulation	96(35.04)	25(23.58)		
Both	14(5.11)	7(6.60)		
Medication use history, n (%)				
Previous antiplatelet	36(13.14)	12(11.32)	0.229	0.632
Previous statin	25(9.12)	9(8.49)	0.038	0.846
Previous antihypertension	158(57.67)	58(54.72)	0.271	0.603
Previous hypoglycemic agents	43(15.69)	15(14.15)	0.297	0.586
Neuropsychological evaluation				
NIHSS score, median (IQR)	1(1–3)	2(1–4)	-2.860	0.004
mRS score, median (IQR)	1(1–2)	2(1–3)	-3.663	<0.001
BI score, median (IQR)	95(75–100)	75(45–100)	-4.443	<0.001
HAMD-17score, median (IQR)	5(3–5)	11(8–15)	-14.787	<0.001
laboratory data				
WBC (×10^9^/L)	6.87 ± 1.88	6.68 ± 1.86	0.883	0.378
Neutrophils (×10^9^/L)	4.49 ± 1.64	5.57 ± 1.83	-0.584	0.559
Lymphocytes (×10^9^/L)	1.76 ± 0.68	1.61 ± 0.66	1.899	0.058
Cr (µmol/L)	71.76 ± 21.64	75.95 ± 38.81	-1.320	0.188
UA (µmol/L)	341.27 ± 81.87	328.27 ± 98.12	1.177	0.691
TG (mmol/L)	2.14 ± 2.09	2.51 ± 9.04	-0.398	0.496
TC (mmol/L)	4.55 ± 1.44	4.28 ± 1.08	1.923	0.055
HDL-C(mmol/L)	1.02 ± 0.30	1.02 ± 0.26	-0.071	0.944
LDL-C(mmol/L)	2.69 ± 0.96	2.51 ± 0.85	1.743	0.082
Fibrinogen (g/L)	2.4(2.2–2.8)	2.9(2.4–3.5)	-5.858	<0.001
Hcy (µmol/L)	9.55(6.88–10.78)	11.8(10–14)	-7.498	<0.001

NIHSS, National Institutes of Health Stroke Scale; mRS, modified Rankin Scale; BI, Barthel Index; HAMD-17, Hamilton depression scale 17 items; WBC, white blood cell; Hcy, homocysteine; HDL-C, high-density lipoprotein cholesterol; IQR, interquartile range; LDL-C, low-density lipoprotein cholesterol; TC, total cholesterol; TG, triglyceride; Cr, creatinine; UA, Uric acid. Values are shown as number (percentage) or as medians (IQR) and mean (SD). Differences in characteristics between groups were analyzed using one-way ANOVA test or Mann-Whitney U test for continuous variables as well as the chi-squared test or Fisher’s exact test for categorical variables. A two-tailed value of P<0.05 was considered significant.

### Logistic regression analysis for risk factors with early-onset PSD

**Figure 4:**
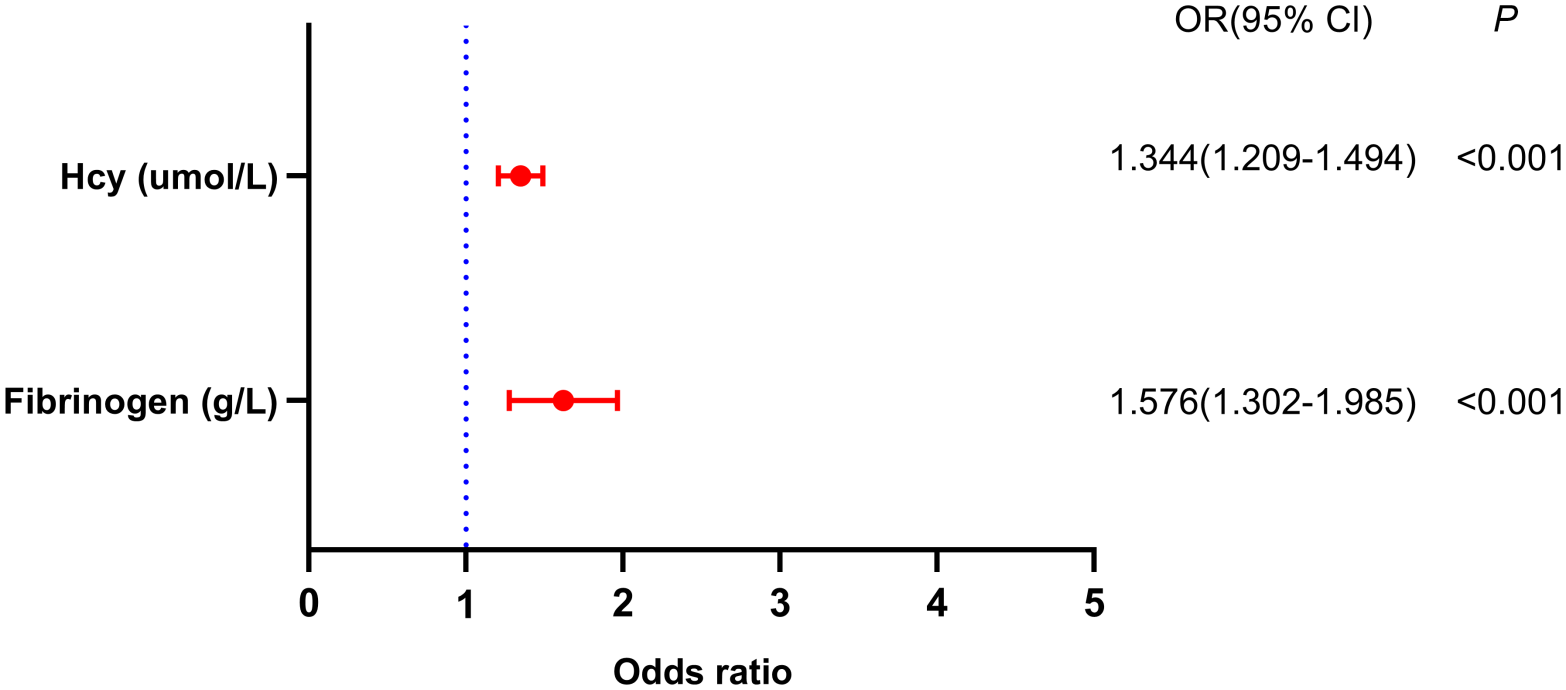
Binary logistic analysis of independent variables associated with early-onset PSD.

Logistic regression models were used to examine the risk factors associated with early-onset PSD. **Table 2** illustrates the results of crude models for early-onset PSD. Crude models showed that gender, current smoking, NIHSS score, mRS score, BI score, Hcy, and fibrinogen were associated with early-onset PSD (P<0.05). In addition, increased Hcy level depends on age. We classified age as a binary categorical variable. After adjustment for all potential confounders, fibrinogen (OR, 1.576; 95% CI 1.302–1.985, P<0.001) and Hcy (OR, 1.344; 95% CI 1.209–1.494, P<0.001) were identified as independent factors for early-onset PSD (**Figure 4**).

**Table 2:** Logistic regression analysis for risk factors associated with early-onset PSD.

Variable	OR (95% CI)	*P*	Adjusted OR (95% CI)	*P*
Age < 65 years	Reference		Reference	
≥ 65 years	1.342(1.136–1.571)	0.135	1.012(0.993–1.032)	0.213
gender	0.561(0.329–0.957)	0.034	0.698(0.359–1.356)	0.288
Current smoking	0.585(0.368–0.929)	0.023	0.589(0.323–1.074)	0.084
NIHSS score	1.215(1.076–1.373)	0.002	1.001(0.812–1.235)	0.992
mRS score	1.483(0.840–1.632)	0.004	1.487(0.941–2.349)	0.089
BI score	1.142(1.041–1.255)	0.005	1.003(0.989–1.017)	0.523
fibrinogen	1.626(1.412–1.998)	<0.001	1.576(1.302–1.985)	<0.001
Hcy	1.405(1.270–1.555)	<0.001	1.344(1.209–1.494)	<0.001

### ROC curves for the Hcy and fibrinogen were used to test the overall discriminative ability for early-onset PSD

**Figure 5:**
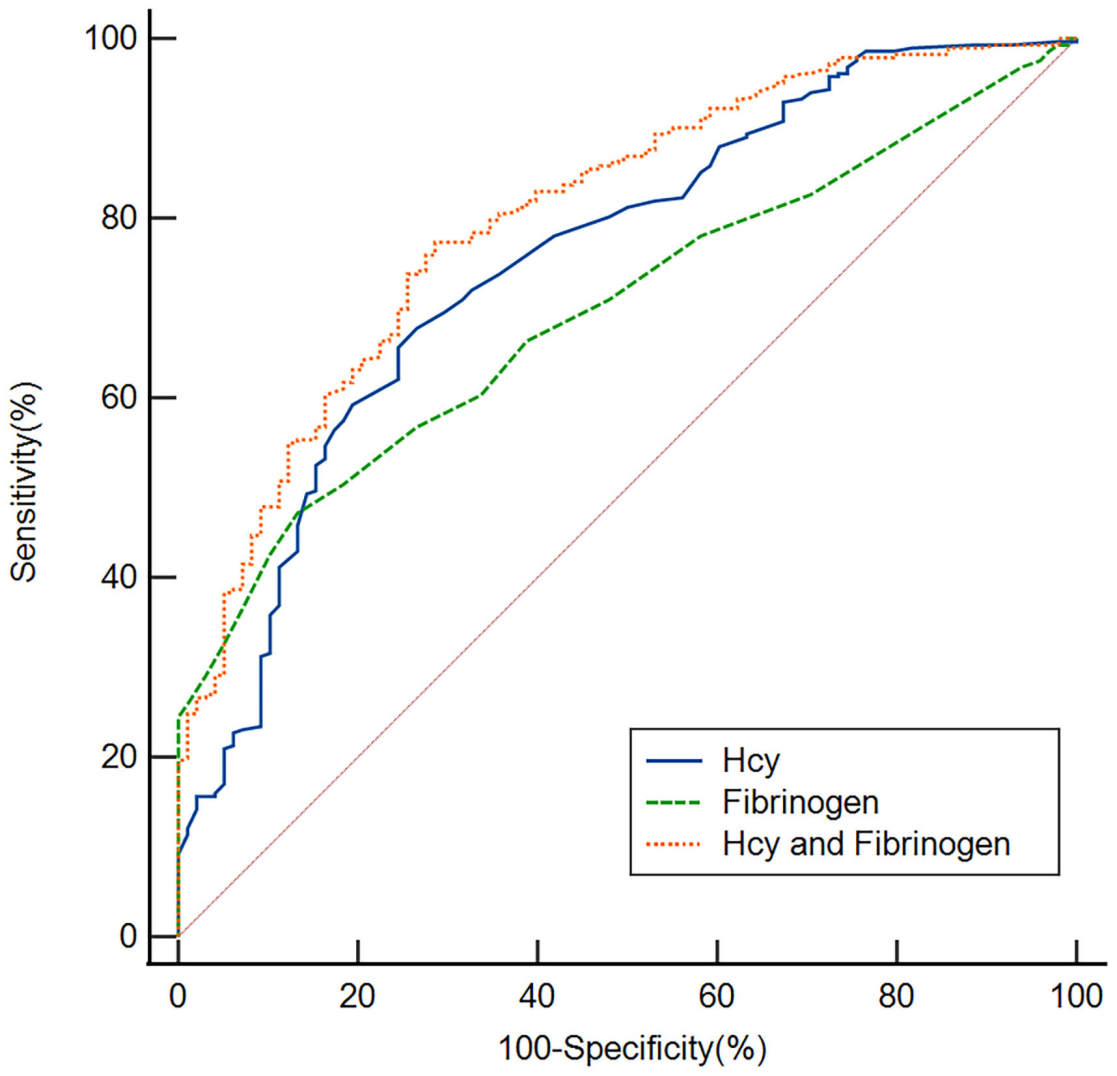
Based on ROC analysis, the Hcy, fibrinogen and Hcy combine fibrinogen exhibited respectable early-onset PSD discriminating power with AUC values of 0.754, 0.698 and 0.803, respectively.

We employed ROC curves to test the overall discriminatory ability of Hcy and fibrinogen for early-onset PSD (**Figure 5**). We observed that the area under the curve (AUC) of the Hcy level to discriminate early-onset PSD was 0.754 (95% CI, 0.708–0.797, P<0.001), the optimal cutoff was 10.55, and the sensitivity and specificity were 67.7% and 73.5%, respectively. The AUC of the fibrinogen was 0.698 (95% CI, 0.649–0.744, P<0.001), the cutoff was 2.95, and the sensitivity and specificity were 47.2% and 86.7%, respectively. In addition, we also conducted an ROC analysis for the combination of Hcy and fibrinogen levels in discriminating between non-PSD and early-onset PSD. The AUC for Hcy and fibrinogen was 0.803 (95% CI: 0.759–0.842, P<0.001), the optimal cutoff was 0.69, and the sensitivity and specificity were 77.3% and 71.4%, respectively.

## Discussion

Our findings provide several novel observations. First, patients in the early-onset PSD group had higher Hcy, fibrinogen, NIHSS scores and mRS scores and a lower BI score than patients in the non-PSD group. Second, the logistic regression model indicated that Hcy and fibrinogen were independent factors for early-onset PSD. Finally, based on ROC analysis, the Hcy combined fibrinogen exhibited respectable early-onset PSD discriminating power. Together, these data support the hypothesis that Hcy and fibrinogen were associated with early-onset PSD.

In our study, 27.89% of the patients were diagnosed with early-onset PSD at 2 weeks after stroke onset. Similar to our results, a meta-analysis reported that approximately 31% of stroke survivors were found to have depression at any time-point up to 5 years after stroke (36). We discovered that the early-onset PSD group showed a higher NIHSS and mRS score and a lower BI score compared to the non-PSD group, which indicated the higher severity of disability and stroke in the early-onset PSD group. Adverse physical conditions may be stressors for developing psychological problems like depression (37). Previous studies have shown that Hcy level increased with age (38). We classified age as a binary categorical variable, and after adjustment for all potential confounders, we discovered that serum levels of Hcy were independently related to early-onset PSD. The result indicated that high serum levels of Hcy in the acute phase of ischemic stroke may be a risk factor for early-onset PSD. Elevated serum Hcy levels at admission were associated with depression at 2 weeks and 6 months after stroke onset (18, 39), which is consistent with our findings. In addition, Hcy has been associated with depressive symptoms 1 year after stroke in older Swedish adults (40). Previous studies have shown that stroke patients with higher Hcy and lower folate levels may be more susceptible to PSD (41). A meta-analysis reports that serum Hcy level can be used as a biomarker to predict the risk of early-onset PSD (42). In our study, the Hcy exhibited respectable early-onset PSD discriminating power with AUC values of 0.754, this is consistent with previous studies (43).

Previous studies have proposed that there are several factors influencing Hcy levels, such as age, sex, BMI, smoking, coffee drinking, poor nutrition, vitamin intake, folate, and ultraviolet radiation (44, 45). In our study, there was no significant correlation between age and Hcy. In addition, genetic variants in the folate and methionine cycles affect males and menopausal women differently (46). However, there was no significant relationship between Hcy concentrations and sex in our study. We believe that the variances in the ethnicity of the research populations, the small sample sizes, the medication status, and the severity of the condition may be the causes of the discrepancies between various studies.

The role of Hcy in early-onset PSD may involve multiple mechanisms. Firstly, an increase in Hcy levels can lead to a reduction in S-adenosylmethionine synthesis in the methionine cycle, which leads to depression (47). Secondly, hyperhomocysteinemia is toxic to nerve cells and endothelial cells. High serum Hcy levels cause mitochondrial dysfunction, leading to neuronal damage in the ischemic cerebral cortex and hippocampus of rats (48). Hcy can aggravate depression-like disorders in post-stroke rats (49). Meanwhile, Hcy exerts a neurotoxic effect on hippocampal neuronal cells by regulating ionic glutamate receptors and inducing apoptosis in hippocampal neurons (50), which promotes the occurrence and development of depression. Thirdly, elevated Hcy levels lead to vascular endothelial dysfunction (51) and an inflammatory response (52). Depression has been associated to the emergence and progression of proinflammatory cytokines, vascular endothelial cell injury and death, and disruption of the blood brain barrier (53, 54). Furthermore, a strong association between Hcy level and inflammation has been reported in various studies (9, 10), and inflammatory processes have been implicated in the pathophysiology of depression (55).

As a type of plasmatic coagulation factor and inflammatory marker, increased fibrinogen concentrations are common in ischemic stroke patients and associated with psychological distress (26). In our research, it was shown that fibrinogen levels were positively correlated with early-onset PSD severity. Furthermore, after adjustment for confounding factors, we found that serum fibrinogen levels were independently associated with early-onset PSD, consistent with previous findings (26, 28, 43). In this study, the AUC of the fibrinogen was 0.698 and may be used as a predictive indicator for early-onset PSD. Although the underlying mechanism by which elevated fibrinogen levels cause post-stroke depressive symptomology remains inconclusive, accumulating evidence supports the role of fibrinogen in the inflammatory response (56). Fibrinogen can increase during any inflammatory event and serves to control systemic inflammatory signals (57, 58). According to a study, fibrinogen might accumulate in inflammatory foci, and extravascular deposits would make inflammation worse (56). Moreover, *in vitro* experiments revealed the important role of fibrinogen in driving inflammation and identified the mechanism by which fibrinogen controls leukocyte function (56). Additionally, several studies have suggested that fibrinogen might be involved in the expression of proinflammatory cytokines IL-6, IL-1β, TNF-α, and IFN-γ (59, 60), and these inflammatory factors are involved in the pathogenesis of PSD (8).

In summary, our study indicates that Hcy and fibrinogen were associated with early-onset PSD. The results of our ROC curve analysis showed that the Hcy and fibrinogen levels had appropriate sensitivity and specificity to discriminate early-onset PSD. Clearly, the Hcy level is more discriminating than the fibrinogen, suggesting that the Hcy level at admission may be a useful tool to predict early-onset PSD. Finally, we found that the combination of Hcy and fibrinogen had a better ability to discriminate early-onset PSD with an AUC of 0.803, suggesting the combination of these two markers has a greater value in predicting early-onset PSD.

This study has the following limitations: (1) it was a single-center study with a relatively small sample size; therefore, the study findings still need to be further confirmed by multi-center and large-sample clinical studies; (2) patients with severe aphasia, unconsciousness, or dementia during hospitalization were excluded from the research, which would have resulted in biases in the prevalence of early-onset PSD; (3) our study did not include several risk variables that may impact depressive episodes, such as social support, educational background, and increasing life stress; (4) we observed PSD only 2 weeks after stroke onset. Short-term observational study conclusions might not be thorough enough; (5) patients were not tested for the vitamin B and folates levels. Future research needs to consider the effect of vitamins B and folate on Hcy levels; and (6) we did not collect homocysteine metabolism genes, and taking genetic factors into account will be important research in the future. To completely understand how Hcy and fibrinogen levels impact the incidence of early-onset PSD, more clinical research with large-scale, long-term intervention and follow-up is required.

## Conclusion

In conclusion, our study indicated that elevated serum levels of Hcy and fibrinogen may be independent risk factors for early-onset PSD and can be used as predictive indicators for early-onset PSD. The combination of Hcy and fibrinogen may provide greater predictive value. Therapies that reduce serum Hcy and fibrinogen levels may be potential targets for intervention and prevention of early-onset PSD.

## Data availability statement

The raw data supporting the conclusions of this article will be made available by the authors, without undue reservation.

## Ethics statement

The studies involving humans were approved by Ethics Committee of the Affiliated Changsha Central Hospital, Hengyang Medical School, University of South China. The studies were conducted in accordance with the local legislation and institutional requirements. Written informed consent for participation was not required from the participants or the participants’ legal guardians/next of kin because this is a retrospective study. Written informed consent was not obtained from the individual(s) for the publication of any potentially identifiable images or data included in this article because this is a retrospective study.

## Author contributions

FL: Data curation, Formal analysis, Investigation, Methodology, Software, Writing – original draft. KS: Data curation, Formal analysis, Funding acquisition, Writing – review & editing. ZW: Data curation, Project administration, Supervision, Visualization, Writing – review & editing. WZ: Data curation, Investigation, Writing – original draft. JG: Data curation, Investigation, Writing – review & editing. SC: Data curation, Formal analysis, Methodology, Writing – review & editing. YT: Data curation, Validation, Writing – review & editing. WX: Project administration, Software, Supervision, Writing – review & editing. NZ: Conceptualization, Supervision, Validation, Writing – review & editing. MD: Conceptualization, Methodology, Project administration, Supervision, Writing – review & editing.

## References

[1] Medeiros GC Roy D Kontos N Beach SR. Post-stroke depression: A 2020 updated review. Gen Hosp Psychiatry. (2020) 66:70–80. doi: 10.1016/j.genhosppsych.2020.06.011 32717644

[2] Guo J Wang J Sun W Liu X. The advances of post-stroke depression: 2021 update. J Neurol. (2022) 269:1236–49. doi: 10.1007/s00415-021-10597-4 34052887

[3] Zhou J Fangma Y Chen Z Zheng Y. Post-stroke neuropsychiatric complications: types, pathogenesis, and therapeutic intervention. Aging Dis. (2023) 14:2127–52. doi: 10.14336/ad.2023.0310-2 PMC10676799 37199575

[4] Cai W Mueller C Li YJ Shen WD Stewart R. Post stroke depression and risk of stroke recurrence and mortality: A systematic review and meta-analysis. Ageing Res Rev. (2019) 50:102–9. doi: 10.1016/j.arr.2019.01.013 30711712

[5] Huang J Zhou FC Guan B Zhang N Wang A Yu P. Predictors of remission of early-onset poststroke depression and the interaction between depression and cognition during follow-up. Front Psychiatry. (2018) 9:2018.00738. doi: 10.3389/fpsyt.2018.00738 PMC6331416 30670990

[6] Lin W Xiong L Yang Z Deng X Zhu J Chen C. Severe periodontitis is associated with early-onset poststroke depression status. J Stroke Cerebrovasc Dis. (2019) 28:104413. doi: 10.1016/j.jstrokecerebrovasdis.2019.104413 31582272

[7] Zeng YY Wu MX Geng DD Cheng L Zhou SN Fan KL. Early-onset depression in stroke patients: effects on unfavorable outcome 5 years post-stroke. Front Psychiatry. (2021) 12:2021.556981. doi: 10.3389/fpsyt.2021.556981 PMC8267172 34248685

[8] Zhang Y Yang Y Li H Feng Q Ge W Xu X. Investigating the potential mechanisms and therapeutic targets of inflammatory cytokines in post-stroke depression. Mol Neurobiol. (2024) 61:132–47. doi: 10.1007/s12035-023-03563-w 37592185

[9] Álvarez-Sánchez N Álvarez-Ríos AI Guerrero JM García-García FJ Rodríguez-Mañas L Cruz-Chamorro I. Homocysteine and C-reactive protein levels are associated with frailty in older Spaniards: the toledo study for healthy aging. J Gerontol A Biol Sci Med Sci. (2020) 75:1488–94. doi: 10.1093/gerona/glz168 31304964

[10] Fang P Li X Shan H Saredy JJ Cueto R Xia J. Ly6C(+) inflammatory monocyte differentiation partially mediates hyperhomocysteinemia-induced vascular dysfunction in type 2 diabetic db/db mice. Arterioscler Thromb Vasc Biol. (2019) 39:2097–119. doi: 10.1161/atvbaha.119.313138 PMC6761027 31366217

[11] Panova-Noeva M Schulz A Arnold N Hermanns MI Prochaska JH Laubert-Reh D. Coagulation and inflammation in long-term cancer survivors: results from the adult population. J Thromb Haemost. (2018) 16:699–708. doi: 10.1111/jth.13975 29431889

[12] Deng M Song K Tong Y Chen S Xu W He G. Higher fibrinogen and neutrophil-to-lymphocyte ratio are associated with the early poor response to intravenous thrombolysis in acute ischemic stroke. Front Neurol. (2024) 15:2024.1291950. doi: 10.3389/fneur.2024.1291950 PMC10919149 38456149

[13] Moradi F Lotfi K Armin M Clark CCT Askari G Rouhani MH. The association between serum homocysteine and depression: A systematic review and meta-analysis of observational studies. Eur J Clin Invest. (2021) 51:e13486. doi: 10.1111/eci.13486 33423269

[14] Saraswathy KN Ansari SN Kaur G Joshi PC Chandel S. Association of vitamin B12 mediated hyperhomocysteinemia with depression and anxiety disorder: A cross-sectional study among Bhil indigenous population of India. Clin Nutr ESPEN. (2019) 30:199–203. doi: 10.1016/j.clnesp.2019.01.009 30904222

[15] Li Y Cao LL Liu L Qi QD. Serum levels of homocysteine at admission are associated with post-stroke depression in acute ischemic stroke. Neurol Sci. (2017) 38:811–7. doi: 10.1007/s10072-017-2848-2 28215036

[16] Cheng LS Tu WJ Shen Y Zhang LJ Ji K. Combination of high-sensitivity C-reactive protein and homocysteine predicts the post-stroke depression in patients with ischemic stroke. Mol Neurobiol. (2018) 55:2952–8. doi: 10.1007/s12035-017-0549-8 28456936

[17] Yin J Zhong C Zhu Z Bu X Xu T Guo L. Elevated circulating homocysteine and high-sensitivity C-reactive protein jointly predicts post-stroke depression among Chinese patients with acute ischemic stroke. Clin Chim Acta. (2018) 479:132–7. doi: 10.1016/j.cca.2018.01.011 29325799

[18] Zhang J Zeng C Huang X Liao Q Chen H Liu F. Association of homocysteine and polymorphism of methylenetetrahydrofolate reductase with early-onset post stroke depression. Front Nutr. (2022) 9:2022.1078281. doi: 10.3389/fnut.2022.1078281 PMC9763289 36562046

[19] Tsang BL Devine OJ Cordero AM Marchetta CM Mulinare J Mersereau P. Assessing the association between the methylenetetrahydrofolate reductase (MTHFR) 677C>T polymorphism and blood folate concentrations: a systematic review and meta-analysis of trials and observational studies. Am J Clin Nutr. (2015) 101:1286–94. doi: 10.3945/ajcn.114.099994 25788000

[20] Li ZC Huang M Yao QY Lin CH Hong BC Wang JH. Association between MTHFR C677T Gene Polymorphisms and the Efficacy of Vitamin Therapy in lowering Homocysteine Levels among Stroke Patients with Hyperhomocysteinemia. J Integr Neurosci. (2024) 23:3. doi: 10.31083/j.jin2301003 38287840

[21] Qin X Spence JD Li J Zhang Y Li Y Sun N. Interaction of serum vitamin B(12) and folate with MTHFR genotypes on risk of ischemic stroke. Neurology. (2020) 94:e1126–36. doi: 10.1212/wnl.0000000000008932 PMC7220236 31932513

[22] Mei F Wu Y Ding G Pan F Chen L Wu J. Association of methylenetetrahydrofolate reductase gene 677C>T polymorphism with post-stroke depression risk and antidepressant treatment response in Han Chinese. J Pak Med Assoc. (2018) 68:888–92. 30174331

[23] Shimohina NY Petrova MM Savchenko AA Piliugina MS. An effect of valdoxan on the hemostasis in patients with acute coronary syndrome in the combination with anxiety and depressive disorders. Zh Nevrol Psikhiatr Im S S Korsakova. (2015) 115:30–6. doi: 10.17116/jnevro20151152130-36 26081321

[24] Austin AW Wissmann T von Kanel R. Stress and hemostasis: an update. Semin Thromb Hemost. (2013) 39:902–12. doi: 10.1055/s-0033-1357487 24114007

[25] von Känel R Dimsdale JE Adler KA Patterson TL Mills PJ Grant I. Effects of depressive symptoms and anxiety on hemostatic responses to acute mental stress and recovery in the elderly. Psychiatry Res. (2004) 126:253–64. doi: 10.1016/j.psychres.2004.02.003 15157751

[26] Luan X Cheng H Chen Y Cheng L Zhou S Song J. High levels of plasma fibrinogen and prothrombin time are related to post-stroke emotional impairment. Brain Res. (2020) 1748:147017. doi: 10.1016/j.brainres.2020.147017 32681836

[27] Qiu X Wang H Lan Y Miao J Pan C Sun W. Blood biomarkers of post-stroke depression after minor stroke at three months in males and females. BMC Psychiatry. (2022) 22:162. doi: 10.1186/s12888-022-03805-6 35241021 PMC8896360

[28] Sun W Miao J Song Y Wang Y Pan C Li G. Systemic low-grade inflammation and depressive symptomology at chronic phase of ischemic stroke: The chain mediating role of fibrinogen and neutrophil counts. Brain Behav Immun. (2022) 100:332–41. doi: 10.1016/j.bbi.2021.10.011 34728390

[29] Liu F Song M Huang X Yi H Chen H Tian F. Symptomatic plaque enhancement is associated with early-onset post-stroke depression. J Affect Disord. (2022) 306:281–7. doi: 10.1016/j.jad.2022.03.026 35337924

[30] Neurology C Society C. Chinese guidelines for diagnosis and treatment of acute ischemic stroke 2018. Chin J Neurol. (2018) 51:666–82. doi: 10.3760/cma.j.issn.1006-7876.2018.09.004

[31] Battle DE. Diagnostic and statistical manual of mental disorders (DSM). Codas. (2013) 25:191–2. doi: 10.1590/s2317-17822013000200017 24413388

[32] Hamilton M. A rating scale for depression. J Neurol Neurosurg Psychiatry. (1960) 23:56–62. doi: 10.1136/jnnp.23.1.56 14399272 PMC495331

[33] Zhang Y He JR Liang HB Lu WJ Yang GY Liu JR. Diabetes mellitus is associated with late-onset post-stroke depression. J Affect Disord. (2017) 221:222–6. doi: 10.1016/j.jad.2017.06.045 28654846

[34] Shen H Tu X Luan X Zeng Y He J Tang W. Serum lipid profiles and post-stroke depression in acute ischemic stroke patients. Neuropsychiatr Dis Treat. (2019) 15:1573–83. doi: 10.2147/ndt.S204791 PMC6589522 31354274

[35] Zimmerman M Martinez JH Young D Chelminski I Dalrymple K. Severity classification on the hamilton depression rating scale. J Affect Disord. (2013) 150:384–8. doi: 10.1016/j.jad.2013.04.028 23759278

[36] Hackett ML Pickles K. Part I: frequency of depression after stroke: an updated systematic review and meta-analysis of observational studies. Int J Stroke. (2014) 9:1017–25. doi: 10.1111/ijs.12357 25117911

[37] Thabrew H Stasiak K Hetrick SE Donkin L Huss JH Highlander A. Psychological therapies for anxiety and depression in children and adolescents with long-term physical conditions. Cochrane Database Syst Rev. (2018) 12:Cd012488. doi: 10.1002/14651858.CD012488.pub2 30578633 PMC6353208

[38] Zong J Sun Y. Retrospective study to identify homocysteine reference intervals in healthy Chinese 60 years of age and above. J Med Biochem. (2023) 42:630–7. doi: 10.5937/jomb0-40154 PMC10710820 38084248

[39] Tang CZ Zhang YL Wang WS Li WG Shi JP. Serum levels of high-sensitivity c-reactive protein at admission are more strongly associated with poststroke depression in acute ischemic stroke than homocysteine levels. Mol Neurobiol. (2016) 53:2152–60. doi: 10.1007/s12035-015-9186-2 25941076

[40] Pascoe MC Crewther SG Carey LM Noonan K Crewther DP Linden T. Homocysteine as a potential biochemical marker for depression in elderly stroke survivors. Food Nutr Res. (2012) 56. doi: 10.3402/fnr.v56i0.14973 PMC3326342 22509143

[41] Chatterjee K Fall S Barer D. Mood after stroke: a case control study of biochemical, neuro-imaging and socio-economic risk factors for major depression in stroke survivors. BMC Neurol. (2010) 10:125. doi: 10.1186/1471-2377-10-125 21192808 PMC3022771

[42] Chen Y Zou H Peng M Chen Y. Association between homocysteine levels in acute stroke and poststroke depression: A systematic review and meta-analysis. Brain Behav. (2022) 12:e2626. doi: 10.1002/brb3.2626 35605010 PMC9226802

[43] Zhou H Wang C Wang W Li H Hu Q Huang N. Lesion location and serum levels of homocysteine are associated with early-onset post-stroke depression in acute ischemic stroke. Brain Behav. (2023) 13:e3210. doi: 10.1002/brb3.3210 37587778 PMC10570478

[44] Nwanaji-Enwerem JC Colicino E Gao X Wang C Vokonas P Boyer EW. Associations of plasma folate and vitamin B6 with blood DNA methylation age: an analysis of one-carbon metabolites in the VA normative aging study. J Gerontol A Biol Sci Med Sci. (2021) 76:760–9. doi: 10.1093/gerona/glaa257 PMC8355450 33027507

[45] Jones P Lucock M Martin C Thota R Garg M Yates Z. Independent and interactive influences of environmental UVR, vitamin D levels, and folate variant MTHFD1-rs2236225 on homocysteine levels. Nutrients. (2020) 12:1455. doi: 10.3390/nu12051455 32443475 PMC7284830

[46] Dierkes J Jeckel A Ambrosch A Westphal S Luley C Boeing H. Factors explaining the difference of total homocysteine between men and women in the European Investigation Into Cancer and Nutrition Potsdam study. Metabolism. (2001) 50:640–5. doi: 10.1053/meta.2001.23286 11398138

[47] Bhatia P Singh N. Homocysteine excess: delineating the possible mechanism of neurotoxicity and depression. Fundam Clin Pharmacol. (2015) 29:522–8. doi: 10.1111/fcp.12145 26376956

[48] Chen S Dong Z Zhao Y Sai N Wang X Liu H. Homocysteine induces mitochondrial dysfunction involving the crosstalk between oxidative stress and mitochondrial pSTAT3 in rat ischemic brain. Sci Rep. (2017) 7:6932. doi: 10.1038/s41598-017-07112-z 28761070 PMC5537278

[49] Wang M Liang X Zhang Q Luo S Liu H Wang X. Homocysteine can aggravate depressive like behaviors in a middle cerebral artery occlusion/reperfusion rat model: a possible role for NMDARs-mediated synaptic alterations. Nutr Neurosci. (2023) 26:483–95. doi: 10.1080/1028415x.2022.2060642 35416761

[50] Kang J Wang D Duan Y Zhai L Shi L Guo F. Aerobic exercise prevents depression via alleviating hippocampus injury in chronic stressed depression rats. Brain Sci. (2020) 11:9. doi: 10.3390/brainsci11010009 33374661 PMC7822431

[51] Jiang X Yang F Tan H Liao D Bryan RM Jr. Randhawa JK. Hyperhomocystinemia impairs endothelial function and eNOS activity via PKC activation. Arterioscler Thromb Vasc Biol. (2005) 25:2515–21. doi: 10.1161/01.ATV.0000189559.87328.e4 PMC4400833 16210565

[52] Kumar M Sandhir R. Hydrogen sulfide suppresses homocysteine-induced glial activation and inflammatory response. Nitric Oxide. (2019) 90:15–28. doi: 10.1016/j.niox.2019.05.008 31146011

[53] Esse R Barroso M Tavares de Almeida I Castro R. The contribution of homocysteine metabolism disruption to endothelial dysfunction: state-of-the-art. Int J Mol Sci. (2019) 20:867. doi: 10.3390/ijms20040867 30781581 PMC6412520

[54] Hayley S Hakim AM Albert PR. Depression, dementia and immune dysregulation. Brain. (2021) 144:746–60. doi: 10.1093/brain/awaa405 PMC8041341 33279966

[55] Frank D Gruenbaum BF Zlotnik A Semyonov M Frenkel A Boyko M. Pathophysiology and current drug treatments for post-stroke depression: A review. Int J Mol Sci. (2022) 23:15114. doi: 10.3390/ijms232315114 36499434 PMC9738261

[56] Luyendyk JP Schoenecker JG Flick MJ. The multifaceted role of fibrinogen in tissue injury and inflammation. Blood. (2019) 133:511–20. doi: 10.1182/blood-2018-07-818211 PMC6367649 30523120

[57] Huang W Wang S Zhang H Zhang B Wang C. Prognostic significance of combined fibrinogen concentration and neutrophil-to-lymphocyte ratio in patients with resectable non-small cell lung cancer. Cancer Biol Med. (2018) 15:88–96. doi: 10.20892/j.issn.2095-3941.2017.0124 29545972 PMC5842339

[58] Ubaldo OGV Lazaro MAE Aventura ET Cinco JE. Can serum fibrinogen predict ARDS? Infect Dis (Auckl). (2020) 13:1178633720943505. doi: 10.1177/1178633720943505 32733125 PMC7372612

[59] Flick MJ LaJeunesse CM Talmage KE Witte DP Palumbo JS Pinkerton MD. Fibrin(ogen) exacerbates inflammatory joint disease through a mechanism linked to the integrin alphaMbeta2 binding motif. J Clin Invest. (2007) 117:3224–35. doi: 10.1172/jci30134 PMC2000806 17932565

[60] Steinbrecher KA Horowitz NA Blevins EA Barney KA Shaw MA Harmel-Laws E. Colitis-associated cancer is dependent on the interplay between the hemostatic and inflammatory systems and supported by integrin alpha(M)beta(2) engagement of fibrinogen. Cancer Res. (2010) 70:2634–43. doi: 10.1158/0008-5472.Can-09-3465 PMC4288842 20233870

