# Supplementary material for: Higher homocysteine and fibrinogen are associated with early-onset post-stroke depression in patients with acute ischemic stroke
Source: Front Psychiatry. 2024 Jun 28;15:1371578. doi: 10.3389/fpsyt.2024.1371578 (PMC11239383; doi:10.3389/fpsyt.2024.1371578)
Supplement: Supplementary file 1 [file Table_1.docx]

**Supplementary Table 1**  **Comparison of blood indexes between male and female in patients.**

| **Variable** | **Male(n=281)** | **Female(n=99)** | **T/Z** | ***P*** |
| --- | --- | --- | --- | --- |
| WBC (×10^9^/L) | 6.71±1.79 | 6.74±2.03 | 0.122 | 0.903 |
| Neutrophils (×10^9^/L) | 4.50±1.58 | 4.52±1.60 | 1.460 | 0.145 |
| Lymphocytes (×10^9^/L)  Cr (µmol/L) | 1.61±0.67  80.57±34.30 | 1.73±0.68  62.22±34.03 | 1.634  -4.837 | 0.103  <0.001 |
| UA (µmol/L) | 346.16±97.25 | 299.32±78.49 | -4.600 | <0.001 |
| TG (mmol/L) | 2.59±1.36 | 2.03±1.32 | -0.652 | 0.515 |
| TC (mmol/L) | 4.26±1.07 | 4.55±1.38 | 2.241 | 0.026 |
| HDL-C(mmol/L) | 0.98±0.25 | 1.11±0.29 | 4.463 | <0.001 |
| LDL-C(mmol/L) | 2.53±0.81 | 2.62±1.03 | 0.933 | 0.351 |
| Fibrinogen (g/L)  Hcy (µmol/L) | 2.7(2.3-3.2)  11(9.5-13.1) | 2.8(2.4-3.3)  11(9.2-13.9) | 1.341  0.727 | 0.181  0.468 |
