# Supplementary material for: Higher homocysteine and fibrinogen are associated with early-onset post-stroke depression in patients with acute ischemic stroke
Source: Front Psychiatry. 2024 Jun 28;15:1371578. doi: 10.3389/fpsyt.2024.1371578 (PMC11239383; doi:10.3389/fpsyt.2024.1371578)
Supplement: Supplementary file 2 [file Table_2.docx]

**Supplementary Table 2** **Comparisons of blood indexes between <65 years and ≥ 65 years in patients.**

| **Variable** | **<65 years (n=151)** | **≥ 65 years (n=229)** | **T/Z** | ***P*** |
| --- | --- | --- | --- | --- |
| WBC (×10^9^/L) | 7.00±1.99 | 6.56±1.74 | 2.292 | 0.022 |
| Neutrophils (×10^9^/L) | 5.59±1.72 | 4.46±1.64 | 1.284 | 0.200 |
| Lymphocytes (×10^9^/L)  Cr (µmol/L) | 1.85±0.72  71.47±27.42 | 1.51±0.60  77.19±39.58 | 4.916  -1.547 | <0.001  0.123 |
| UA (µmol/L) | 338.50±91.69 | 327.54±96.15 | 1.106 | 0.270 |
| TG (mmol/L) | 4.69±1.32 | 1.70±1.10 | 2.206 | 0.028 |
| TC (mmol/L) | 4.70±1.32 | 4.12±1.03 | 4.760 | <0.001 |
| HDL-C(mmol/L) | 0.99±0.27 | 1.04±0.27 | -1.840 | 0.067 |
| LDL-C(mmol/L) | 2.83±0.89 | 2.40±0.84 | 5.012 | <0.001 |
| Fibrinogen (g/L)  Hcy (µmol/L) | 2.7(2.3-3.3)  11(9.4-13.2) | 2.8(2.3-3.2)  11.1(9.4-13.6) | 0.508  1.099 | 0.612  0.273 |
